# Is Burning Mouth Syndrome Associated with Extraoral Dryness? A Systematic Review

**DOI:** 10.3390/jcm12206525

**Published:** 2023-10-14

**Authors:** Kim Devon Terga Le, Marcos Fabio DosSantos, Parisa Gazerani

**Affiliations:** 1 Department of Life Sciences and Health, Faculty of Health Sciences, Oslo Metropolitan University, 0130 Oslo, Norway; 2 Laboratório de Propriedades Mecânicas e Biologia Celular (PropBio), Departamento de Prótese e Materiai Dentários, Faculdade de Odontologia, Universidade Federal do Rio de Janeiro (UFRJ), Rio de Janeiro 21941-617, RJ, Brazil; 3 Laboratório de Morfogênese Celular (LMC), Instituto de Ciências Biomédicas (ICB), Universidade Federal do Rio de Janeiro (UFRJ), Rio de Janeiro 21941-590, RJ, Brazil; 4 Programa de Pós-Graduação em Neurociência Translacional, Instituto Nacional de Neurociência Translacional (INNT-UFRJ), Rio de Janeiro 21941-590, RJ, Brazil; 5 Programa de Pós-Graduação em Odontologia (PPGO), Universidade Federal do Rio de Janeiro (UFRJ), Rio de Janeiro 21941-617, RJ, Brazil; 6 Centre for Intelligent Musculoskeletal Health (CIM), Faculty of Health Sciences, Oslo Metropolitan University, 0130 Oslo, Norway; 7 Department of Health Science and Technology, Faculty of Medicine, Aalborg University, 9260 Gistrup, Denmark

**Keywords:** burning mouth syndrome, dryness, oral, extraoral

## Abstract

Burning mouth syndrome (BMS) is characterized by a persistent intraoral burning sensation, often experienced by postmenopausal women. The etiology of BMS remains partially understood, and consequently, treatments remain suboptimal. Extraoral manifestations of BMS, such as extraoral dryness, are less studied. However, it has been suggested that the identification of the frequency and patterns of extraoral dryness and potential underlying mechanisms are essential to optimize treatment strategies and reduce the burden of disease. Therefore, we conducted this systematic review to provide existing evidence about extraoral dryness in BMS. The guidelines for the conduction and reporting of systematic reviews were followed. An electronic search was conducted in four major databases—PubMed, Web of Science, COCHRANE Library, and EBSCOhost—and the grey literature was assessed through Google Scholar. From each included article, information on extraoral dryness in BMS was extracted, and odds ratios were calculated for extraoral dryness among BMS patients compared with non-BMS controls. The findings demonstrated higher odds of the prevalence of extraoral dryness in BMS, which was found to a high degree in the lips, eyes, skin, and genitalia. The pattern of spread and locations of extraoral dryness propose a potential central mechanism. Based on our findings, we encourage the standardization of the assessment, recording, and reporting of the extraoral characteristics of BMS, including extraoral dryness, which can lead to better management strategies and enhance the quality of life of the affected patients.

## 1. Introduction

Oral health is an important public health issue [1]. According to the World Health Organization’s report from 2022, oral diseases affect about 3.5 billion individuals, which accounts for about half of the world’s population [2]. Oral disorders can dramatically reduce the quality of life [3] and increase the risk of developing or worsening other disorders, including somatic [4] and mental disorders [5]. Burning mouth syndrome (BMS) is a chronic, debilitating condition characterized by a persistent burning sensation [6]. The International Classification of Headache Disorders, 3rd edition (IHS) defines BMS as an intraoral burning or dysesthesia sensation recurring daily for over 2 h for at least three months without clinically evident causative lesions [7].

The absence of obvious causative pathosis and the convoluted diagnostic criteria of BMS have left it problematic to diagnose clinically, and it is often a diagnosis of exclusion by the process of elimination [8,9]. The sensation of a burning mouth can be attributed to either local (e.g., hyposalivation, candidiasis, lichen planus, or oral parafunction) or systemic (medication-induced diabetes, anemia, deficiencies of vitamin B12 or folic acid, or Sjögren’s syndrome) factors. In these situations, addressing the underlying cause leads to resolving the burning mouth symptom, and a diagnosis of BMS cannot be established [10]. However, the classification and nomenclature used for BMS still vary among different authors. Therefore, a consensus on disease definition and the diagnostic criteria to be used for randomized clinical trials has been encouraged [11]. The affected oral mucosal areas are usually reported on the tongue, lips, and hard and soft palate [12]. The prevalence of BMS has been reported to be between 0.01% and 40% of the population [13]. A recent study provided a subgroup analysis which showed that the disease prevalence in Asia is 1.05%, in Europe, it was found to be 5.58%, and in North America, it was found to be 1.10% [14]. BMS can occur in individuals in any age group, but the condition predominantly affects individuals in their fifth or later decades of age [15]. A prevalence analysis based on age showed a prevalence rate of 3.31% for people over 50 years old, while it was 1.92% for those under 50 [14]. BMS has been reported to occur in both females and males, but the female-to-male ratio of reported prevalence between the sexes varies from 3:1 to 16:1, which ultimately means that the condition predominantly affects females [16]. Considering gender, a subgroup analysis showed a prevalence of 1.15% in females and a prevalence of 0.38% in males in the general population [14]. The most affected are peri- and postmenopausal women [12]. Despite the prevalence and reported significant impact on patients’ quality of life [17], there is still a lack of understanding about BMS’s etiology and pathophysiology, making the condition challenging to diagnose and manage effectively [6]. Understanding the underlying mechanisms and identifying contributing bio-psycho-social factors [18] will facilitate the development of evidence-based treatment. BMS has been reported to be associated with multiple comorbidities, such as thyroid disorders, chronic fatigue, and gastroesophageal reflux disease [12,19], among extraoral symptoms [4], which could reflect on a more systemic condition [20]. An association has been reported between psychological disorders such as anxiety and depression and BMS [21]. Lower self-esteem, higher vulnerability, and more impulsive decisions among patients with BMS have also been reported [22,23]. BMS accompanies changes in eating habits, altered sleep patterns, and decreased desire to socialize and is often associated with overall negative effects on well-being and poor quality of life [24].

Extraoral dryness has also been reported as a co-existing condition in BMS patients in older [25] and newer [26] studies. Acharya et al. in 2018 presented an association between BMS and self-reported skin conditions, including rosacea, eczema, dry skin, psoriasis, and dryness in the genital area [26], in addition to oral dryness. The same group of researchers also analyzed the salivary samples of BMS patients and controls and demonstrated a complex pattern of inflammatory biomarkers, including Cystatin-5, (CST-5), Interleukin-7 (IL-7), Fibroblast Growth Factor-19 (FGF-19), and C–C Motif Chemokine 19 (CCL-19). Both higher and lower levels of biomarkers were found in the BMS patients compared with the controls, indicating that BMS patients represent a diverse group when considering their levels of inflammatory biomarkers [27]. Although this study provided valuable insights, the concept of extraoral dryness, potential reasons, consequences for patients, and potential strategies to prevent or treat it remain uninvestigated. Therefore, we aimed to conduct a systematic review to identify the existing knowledge within this area. We hypothesized that we would identify studies that have reported dryness inside the oral cavity, outside the oral cavity, and in remote body areas. In addition, we expected that we would find a range of studies that might have linked the pain–dryness cycle aligned with BMS as a symptom, or dryness as a comorbid condition to BMS, regardless of the link to pain, and more as a general body response, for example, at the level of immune system response. The ultimate goal is to use the outcome of this systematic review to form the research agenda for the identification, prevention, and treatment of extraoral dryness in BMS. However, we expect that this systematic review could also contribute to patient awareness and education and guide clinicians toward a more holistic approach to intra- and extraoral dryness in BMS. Realizing the extraoral manifestations in BMS may aid in updating the clinical manifestation and diagnostic criteria. The ultimate goal is to improve care and quality of life for the affected patients.

## 2. Materials and Methods

A systematic review was chosen as the method for conducting this review because this comprehensive and standard method is known to be reliable for summarizing existing evidence thoroughly and transparently, whereby the available evidence is identified, analyzed, and reported [28,29]. The literature was searched in the following major databases: PubMed, Web of Science, COCHRANE Library, and EBSCOhost. Google Scholar was used to assess the grey literature. The choice of these databases was based on the recommendation by Bramer et al. for optimal database combinations in conducting a systematic review [30]. The search strategy was different between the databases but pertained to BMS and comorbidities. The inclusion and exclusion criteria used to select the studies were based on the population, study design, and outcomes regarding BMS and extraoral dryness. Among the various tools for the quality assessment of the included studies [31], the quality assessment tools developed by the National Heart, Lung, and Blood Institute (NHLBI) were used to evaluate the studies. The web-based tool Rayyan was used to organize the identified studies. In the following section, the step-by-step process for conducting this systematic review is presented.

### 2.1. Study Protocol and Registration

It has been recommended to create a systematic review protocol and register it before the actual review phase [28,29]. To prepare the protocol and execute the systematic review, we followed the Preferred Reporting Items for Systematic Reviews and Meta-Analyses (PRISMA) statement checklist and utilized the PRISMA flow diagram [32,33]. Following the preparation of the review protocol, it was registered in the PROSPERO (Prospective Register of Systematic Reviews), which is a systematic review registration database to provide transparent protocols of systematic reviews and avoid waste and unnecessary duplication [34]. The protocol was registered on 7 October 2022 and was then evaluated by the PROSPERO expert team to check for submission eligibility. One main eligibility criterion is that no prior systematic review must be registered or ongoing with a similar or close review question. On 18 October 2022, our systematic review protocol was accepted and registered in the database with the identification number CRD42022365674.

### 2.2. Search Strategy

The PICO (patient/population, intervention, comparison, and outcomes) process was used to develop the clinical review question and the search strategy [35]. The intervention, however, did not apply to this review. We initially added time (T) as this has also been recommended [36], but we found it very limiting after performing a quick preliminary search on Google Scholar and PubMed. Therefore, there was no limit of time for this review to allow us to include all available records. Table 1 presents the PICO setting for this review.

### 2.3. Literature Search

We utilized a well-designed search strategy to minimize the risk of missing important studies and ensure a comprehensive and unbiased synthesis of available evidence. The literature search in Google Scholar, PubMed, Web of Science, COCHRANE Library, and EBSCOhost started on 18 October 2022 and was finalized on 14 November 2022. The searches through the databases were conducted in the same chronological order as listed. The advanced search was utilized if the function was available, i.e., for the case of PubMed with PubMed Advanced Search Builder and MeSH (Medical Subject Headings) terms, Web of Science with Advanced Search Query Builder in their Core Collection, COCHRANE Library with Advanced Search and MeSH, and EBSCOhost with their default Advanced Search option. EBSCOhost provides the option to search through specific databases that are included in the EBSCOhost database, namely, Academic Search Ultimate, CINAHL, eBook Collection, eBook Open Access Collection, Education Source, and MEDLINE, which were included in the EBSCOhost search.

The search strategies in the selected databases were different in their techniques for how to narrow down and focus the search results regarding the utilization of Boolean operators and truncations. Boolean operators are used as conjunctions to combine or exclude keywords, with AND, OR, and NOT as the main Boolean operators in most databases. Truncations are used to shorten a word by including a “*” and will include any endings or spelling of that word, such as for the truncated word “comorbid*” which returns the results for the words comorbid, comorbidity, and comorbidities. Different combinations of these techniques were utilized. A supplementary manual search was also performed during January 2023 to include additional relevant studies.

The number of studies to be identified in a systematic review is not standardized, and the number of studies depends on the research area and research question. However, it is recommended to identify and synthesize all available evidence of the research interest that meets the inclusion criteria regardless of the number [37]. Therefore, we attempted to include available evidence with no limit of time.

#### 2.3.1. Inclusion and Exclusion Criteria

The main target patient consists of women of pre- and postmenopausal age with BMS. Only studies with clearly defined characteristics of BMS were included. Given the lack of complete standardization in the diagnostic criteria for BMS, we decided to include various diagnostic criteria that have been utilized. The occurrence of other morbidities in the patient group was taken into consideration. The inclusion of comorbidity studies was deemed important as they may include extraoral dryness. The male population was included only when it was mentioned, and separate data were presented. The age means of the population groups were taken into consideration, and studies with an age mean of above 50 years old were included. Studies with an age mean of under 50 years old were excluded. Studies conducted with children, teens, and young adults were also excluded. The population groups included in this systematic review were, therefore, late “middle-aged adults” or “aged” including both females and males.

Only human clinical studies were included, and animal studies and in vitro studies (e.g., cell and tissue works) related to BMS were excluded. Only quantitative studies published in English were included. Studies published in other languages were excluded, although no specific regions of the world were considered a limitation. Due to limited research in this area with only a few empirical studies, all available data were included.

Only full-text published studies were searched and included. Abstract-only texts, such as conference abstracts, proceedings, editorials, preprints, or manuscripts, and other non-full text research journal studies were excluded. Books and documents were also excluded. Epidemiological studies within disease research for burning mouth syndrome were included. This included observational studies such as cohort studies, case–control studies, panel studies, surveys and questionnaires, and experimental studies such as randomized controlled trials.

There was no limitation to the type of included reviews, and topical, systematic, narrative, scoping reviews and meta-analyses were also included in addition to the original research articles. Comparative studies with a BMS population and a control group were included. No limitation was introduced related to the type of the control group (for example, matched or non-matched).

Studies with BMS as the main topic were screened for relevancy for extraoral dryness. The primary outcome of this review was extraoral dryness in patients with BMS. Studies with data of little relevance were excluded. For example, study data not about the primary target topic of BMS were excluded, as well as data not referring to the prevalence or incidence of extraoral dryness within the study population. Studies with discrete quantitative data regarding the prevalence or incidence of extraoral dryness or the dichotomous outcomes of extraoral dryness within the BMS population were included. Table 2 summarizes the inclusion and exclusion criteria.

#### 2.3.2. Search Terms in the Search Databases

The search terms were tailored to the specific review question and included medical subject headings, free-text terms, and Boolean operators to refine the search. The search terms for the literature search were defined specifically to search for the population and outcome. Therefore, the search terms included BMS and different descriptors of outcomes to reflect the main outcome of this review focus, i.e., extraoral dryness. Both full terms and abbreviations were used, for example, for BMS. Many descriptions of the outcome were searched for to accommodate various forms of records in the literature, such as “Dry skin”, “Dryness”, “Xerosis”, “Dermatitis”, “Skin disease”, “comorbidity”, “association”, and “prevalence”. The two search terms “Xerosis” and “Dermatitis” were also included.

Some databases require one to differentiate between the words in the search term so that the included words do not mix or blend. For example, the division of words provides the foundation of which words are searched for in a search term. This was carried out by using parentheses to divide the words in a search term. For example, the inclusion of the population and outcome of this review was formulated as (Burning mouth syndrome patients) AND (dryness). The dividers were used for search terms in all selected databases other than Google Scholar. This is because the Google Scholar database does not recognize parentheses as dividers.

The search terms included many syntax techniques, such as Boolean operators and truncations. The search terms accommodated the differences between the selected databases and, as such, had different utilizations of Boolean operators and truncations. For example, the most common Boolean operator to include for finding synonyms or similar concepts is the usage of “OR”. The Boolean operator “OR” is usable in all selected databases, but one search term in Google Scholar also included an alternative symbol to represent “OR” by using “|”. The Boolean operator “OR”, as well as “AND”, was widely used in the literature search for all of the selected databases.

It was highly relevant to find the words “comorbidity” and “patient” in many of their different endings, hence the usage of truncation in the search terms. The truncation of the words was represented as “comorbid*” and “patient*” in all selected databases other than Google Scholar. The exception of Google Scholar was made because the database does not recognize truncations, which was not a problem due to their automatic stemming of any complete word. Quotation marks were used to search for the exact relevant phrases. This function was used for all the selected databases. Table 3 depicts the list of search terms used for Google Scholar, PubMed, Web of Science, Cochrane Library, and EBSCOhost.

### 2.4. Management Tool for the Systematic Review

Various tools are available to help organize and manage systematic reviews. One of the assisting tools for conducting systematic reviews is Rayyan, which was used in its web-based version for this systematic review. The literature search results were easily transferrable to Rayyan, where independent reviewers could collaborate on screening and article selection, the discussion of conflicts, and resolving in addition to adding full texts and comments.

### 2.5. Screening of Articles

The screening of titles and abstracts was conducted independently (“blind on” function in Rayyan) by both authors. The screening of articles started on 18 November 2022 and finished on 3 December 2022. Rayyan automatically screens for duplicates and recommends the removal of duplicates. Duplicate studies were therefore removed, and the titles and abstracts were screened against the set of eligibility criteria (inclusion/exclusion). Articles that met the criteria were then included or excluded. Any disagreements between the reviewers for inclusion or exclusion were thereafter resolved through discussions. As a backup to Rayyan, the Excel program was used. The entire process, including all articles, files, discussion notes, etc., was stored in a Microsoft Teams folder dedicated to this systematic review.

### 2.6. Synthesizing the Evidence

The extraction of data from the included articles started on 4 December 2022 and finished on 13 February 2023. Full texts of all of the included studies were read for the data collection, and the extracted information was organized in a systematic format in Microsoft Excel. Following the registered review protocol and agreement between the authors, the main data points to extract were general information about the papers (title, year of publication, authors, and place or country of data collection), the patient population (demographic information), study methodology, and the study outcomes.

The data points for the study methodology were the design of the studies, the type of burning mouth syndrome reported, and the study participants. This includes whether the study used a control group, and additional information regarding the control group was also extracted, such as whether they were sex- and age-matched with the burning mouth syndrome patients. Additional relevant data were extracted based on the study design. For example, the length of follow-up and their details were collected if the study design was a cohort study. We also examined the description of the study participants, which included the number of participants and population groups, the sex of the participants, the mean and median age of the participants, and the diagnostic criteria for BMS.

The data points for the study outcomes were the extraoral outcomes in the BMS patients and the outcomes for the control group if they were included in the study. All study outcomes regarding extraoral dryness were collected together with the information on how the outcome was assessed and measured. The definition of extraoral dryness in the studies was carefully considered, with the importance of an exact description such as “dry” or “dryness”. An exception was considered for skin problems where they were ambiguously described as rosacea, eczema, dry skin, and psoriasis. Other common prevalent comorbidities reported in the included studies were also noted.

### 2.7. Quality Assessment

It is important to assess the quality of the included studies by assessing the risk of bias to ensure the integrity and validity of systematic reviews [38]. Bias has multiple forms (measurement bias, information bias, performance bias, detection bias, attrition bias, and selection bias). Risk of bias assessment reflects on the internal validity of the studies, where a low risk of bias implies good study quality and the high strength of the evidence [38]. The quality assessment phase for our review was conducted independently by both authors from 16 March to 16 April 2023. We used the NHLBI. The set of criteria for this tool depends on the study design under quality review and includes multiple questions. The criteria questions are answered with yes, no, or other (cannot determine/not applicable/not reported), and a score is given which determines a rating of good, fair, or poor. From this package, the Quality Assessment of Case–Control Studies and the Quality Assessment Tool for Case Series Studies were used according to the type of the included studies (please see the Section 3).

For the Quality Assessment of Case–Control Studies, the number of yes answers from 0 to 5 is considered poor, the number of yes answers from 6 to 9 is considered fair, and the number of yes answers from 10 to 12 is considered good. For the Quality Assessment Tool for Case Series Studies, the number of yes answers from 0 to 4 is considered poor, the number of yes answers from 5 to 7 is considered fair, and the number of yes answers with 8 or 9 is considered good. Any disagreement regarding the quality ratings was discussed between the authors, and agreement was reached. Studies with poor quality were excluded from further analysis. A good score rating indicates a low risk of bias and, therefore, valid study results; a fair score rating indicates not having sufficient bias to invalidate the study results, and a poor score rating indicates a significant risk of bias; therefore, the study should be excluded.

### 2.8. Prerequisite Checks for a Meta-Analysis: Data Size and Data Heterogeneity

A meta-analysis is to combine data from several studies for advanced statistical analyses [39]. A prerequisite is to have a sufficient amount of qualified data and that the data can pass the heterogeneity test. We conducted the heterogeneity test on 13 April 2023 to assess the data variability of the included studies. The test of heterogeneity was performed by comparing the extracted data from the included studies. The main comparators were the diagnostic criteria for BMS and control groups, study population, study objectives, and study outcomes. Performing prerequisite checks for a meta-analysis resulted in a no-go for a meta-analysis.

### 2.9. Data Synthesis

When a meta-analysis is not feasible, several methods are available such as narrative synthesis, vote counting, thematic synthesis, and framework synthesis [40]. The choice of method depends on the level of appropriateness and the nature of the included studies, the research question, and the available data [40]. The narrative synthesis of the results used for this review was performed according to the guidelines by Denison et al. [41] to obtain odds ratios and 95% confidence intervals. A prevalence diagram was also created to illustrate the body region and prevalence (%) of reported extraoral dryness.

## 3. Results

The execution of the protocol for this systematic review was successful. The standard steps of conducting a systematic review yielded presentable data. In the following, the PRISMA flow diagram for the literature search, a description of the included studies, a quality assessment of the included studies, and summarized data on characteristics and data values for the within- and between-group comparisons regarding the narrative synthesis are presented. A summary table of the extraoral dryness values presenting the calculated odds ratios and 95% confidence intervals, as well as an extraoral dryness prevalence diagram of the reported extraoral dryness values, are also demonstrated.

### 3.1. Literature Search Results on PRISMA

**Figure 1:**
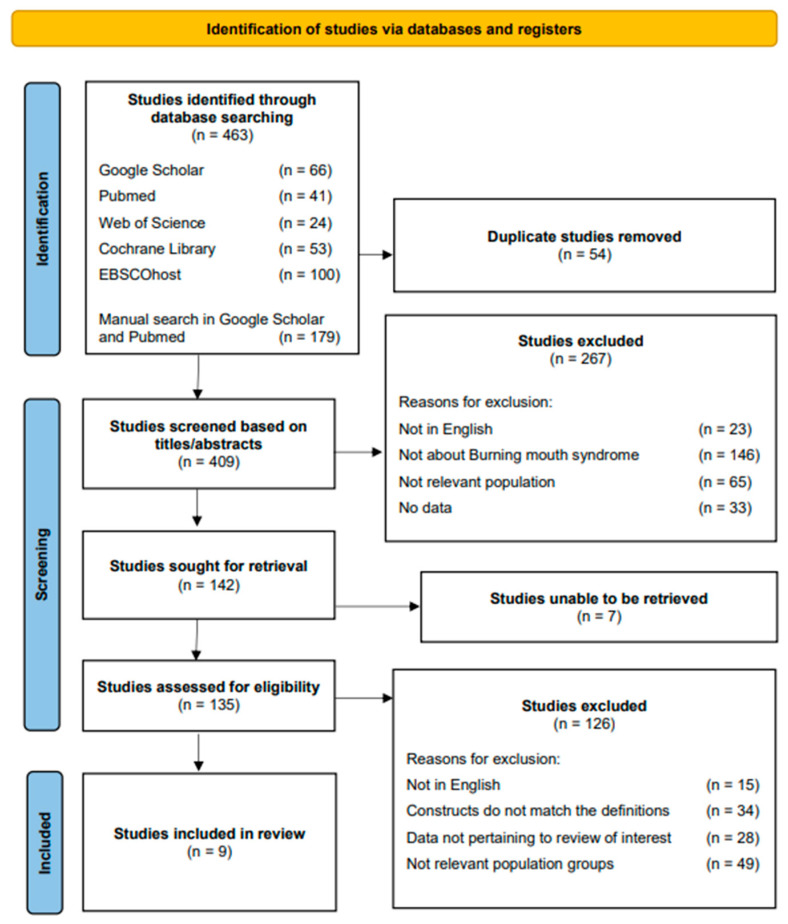
PRISMA flow diagram of the systematic review. The identification stage of this review yielded 463 studies. The number of studies in each stage is listed, and the reasons for exclusion are presented. Arrows are used as a visual guide for the progression. A total of 454 studies were excluded, and the remaining 9 studies were included in the systematic review.

The literature search yielded 284 studies from the electronic databases Google Scholar, PubMed, Web of Science, Cochrane Library, and EBSCOhost. A supplementary manual search yielded an additional 179 studies from the electronic databases Google Scholar and PubMed. The combination of the literature search and the supplementary manual search yielded a total of 463 studies. Following the elimination of duplicates, a total of 409 studies were obtained for screening and potential inclusion in the systematic review. The screening of titles and abstracts for eligibility based on the inclusion and exclusion criteria resulted in the exclusion of 267 studies and the inclusion of 142 studies for the retrieval of the full texts. Seven studies were not retrievable (e.g., no responses from the authors), but 135 full-text studies were collected and assessed based on the inclusion and exclusion criteria for eligibility. This step resulted in the exclusion of 126 studies. The basis for the exclusion was the language used being one other than English, irrelevant constructs such as the design and context, irrelevant data or outcomes, and an irrelevant target population. Following this step, nine studies remained eligible and were included in the next step of the data extraction and narrative synthesis. The PRISMA flow diagram is presented in Figure 1.

### 3.2. Description of the Included Studies

Nine studies were included in this systematic review. The study IDs are presented as Study 1 by Grushka (1987) [25], Study 2 by Lamey et al. (2005) [42], Study 3 by Monteserin-Matesanz et al. (2022) [43], Study 4 by Werfalli et al. (2021) [44], Study 5 by Mignogna et al. (2011) [4], Study 6 by Acharya et al. (2018) [26], Study 7 by De Pedro et al. (2020) [45], Study 8 by Cavalcanti et al. (2007) [46], and Study 9 by Freilich et al. (2020) [47]. As can be seen, the publication date range of the included studies is 1987–2022.

Two types of study designs were identified in the included studies, whereby eight were case–control studies and one was a case series. Seven studies included a control group and presented data from both cases and controls. Five studies were carried out in European countries, three in North American countries, and one in a South American country.

The total number of participants (i.e., cases and controls) varied between the studies and presented a range from 31 to 226 participants (in the order of study ID, the number of participants was identified as 81, 173, 82, 50, 226, 112, 60, 31, and 102). The population consisted mostly of women. The sex between the participant groups of BMS patients and control groups was mostly equal because of the diagnostic criteria for the controls being sex-matched.

The age of the participants between the participant groups was fairly consistent, as the diagnostic criteria of the controls were age-matched. The weighted mean age was calculated and ranged from 57.23 to 67.75 years (in the order of study ID, the mean age was calculated as 57.88, 65, 61.96, 58.64, 57.23, 67.75, 63.62, and 61.3). One study reported a median age of 60 rather than the mean. The background and lifestyles of the patients were not reported in any of the studies.

The diagnostic criteria for burning mouth syndrome varied between the included studies. Table 4 presents the diagnostic criteria used by the included studies. Five studies used a defined classification for BMS diagnosis. Of those, Study 3 [43], Study 6 [26], Study 7 [45], and Study 9 [47] used the Headache Classification Committee of the International Headache Society (IHS) definition of BMS (ICHD-3 clinical definition: 13.11) [7], while Study 4 [25] used the American medical diagnoses and procedure ICD-10-CM code for the definition of BMS (ICD-10-CM diagnosis code K14.6) [48]. Study 1 [25], Study 2 [42], and Study 8 [46] defined the diagnostic criteria of BMS as an oral burning sensation [7]. Of those, Study 1 [25] and Study 8 [46] included a time frame of the persistent effect of the burning sensation, while Study 2 [42] considered only the burning sensation without a time frame. Study 5 [4] reported the diagnostic criteria of BMS as oral pain with a persistent effect in a time frame. BMS classification and BMS types were only reported in one of the studies, Study 9 [47], and were not reported or mentioned in the other studies.

The most common symptom reported was xerostomia, which was reported in six studies. All included studies reported a variety of extraoral dryness in the participants. Other more common symptoms reported in at least three studies were gastrointestinal problems, dysgeusia, and insomnia. The other associated symptoms were less often reported and included dizziness, carcinophobia, and common mental illnesses.

For details, please refer to the summary tables of the results presented in Section 3.4.

### 3.3. Quality Assessment of the Included Studies

Conducting a quality assessment [31] with the NHLBI tool [49] resulted in the quality rating of “Fair” for all included studies. Eight of the studies were case–control studies; therefore, the corresponding Quality Assessment Tool for Case–Control Studies was used. One study was a case series study; therefore, the Quality Assessment Tool for Case Series Studies was used. Table 5 presents the assessment of the risk of bias within the case–control studies. Table 6 presents the assessment of the risk of bias within the case series studies. The case–control quality assessment tool consisted of a set of 12 questions that were answered with Y (yes) and N (no). The case series quality assessment tool consisted of a set of nine questions that were answered with Y (yes) and N (no). The questions are listed in the footnotes of the tables. The assessment allows the number of “Yes” in responses to questions that consequently form the category of poor, fair, or good [49].

The quality assessment tool by the NHLBI [49] was utilized (Quality Assessment of Case–Control Studies). The overall quality scores for the case–control studies are defined by the overall yes answers—poor (0–5), fair (6–9), and good (10–12).

Q1: Was the research question or objective in this paper clearly stated and appropriate? Q2: Was the study population clearly specified and defined? Q3: Did the authors include a sample size justification? Q4: Were the controls selected or recruited from the same or similar population that gave rise to the cases (including the same timeframe)? Q5: Were the definitions, inclusion and exclusion criteria, algorithms, or processes used to identify or select cases and controls valid, reliable, and implemented consistently across all study participants? Q6: Were the cases clearly defined and differentiated from the controls? Q7: If less than 100 percent of eligible cases and/or controls were selected for the study, were the cases and/or controls randomly selected from those eligible? Q8: Was there use of concurrent controls? Q9: Were the investigators able to confirm that the exposure/risk occurred prior to the development of the condition or event that defined a participant as a case? Q10: Were the measures of exposure/risk clearly defined, valid, reliable, and implemented consistently (including the same time period) across all study participants? Q11: Were the assessors of exposure/risk blinded to the case or control status of participants? Q12: Were key potential confounding variables measured and adjusted statistically in the analyses? If matching was used, did the investigators account for matching during the study analysis?

The quality assessment tool by the NHLBI [49] was utilized (Quality Assessment of Case Series Studies). The overall quality scores for the case series studies are defined by the overall yes answers—poor (0–3), fair (4–6), and good (7–9).

Q1: Was the study question or objective clearly stated? Q2: Was the study population clearly and fully described, including a case definition? Q3: Were the cases consecutive? Q4: Were the subjects comparable? Q5: Was the intervention clearly described? Q6: Were the outcome measures clearly defined, valid, reliable, and implemented consistently across all study participants? Q7: Was the length of follow-up adequate? Q8: Were the statistical methods well described? Q9: Were the results well described?

### 3.4. Summary Tables

The findings of this systematic review are presented in summary tables. Table 7 presents the study descriptions, and Table 8 demonstrates the details of the participants in the included studies. Table 9 depicts the primary outcomes of the included studies.

### 3.5. Extraoral Dryness

The extraoral dryness extracted from the included articles was identified as dry eyes, skin problems, genital dryness, and dry lips. The odds ratio and 95% confidence intervals are reported in Table 10. Please note that the calculation did not apply to Study 5 [4], Study 7 [45], Study 8 [46], and Study 9 [47].

The reported extraoral dryness was summed according to the body region (eyes, genital area, skin, and lips), and odds ratio and 95% confidence intervals were calculated if applicable. Table 11 depicts the extraoral dryness summary with the calculated population percentages, odds ratios, and 95% confidence intervals (95% CIs).

**Figure 2:**
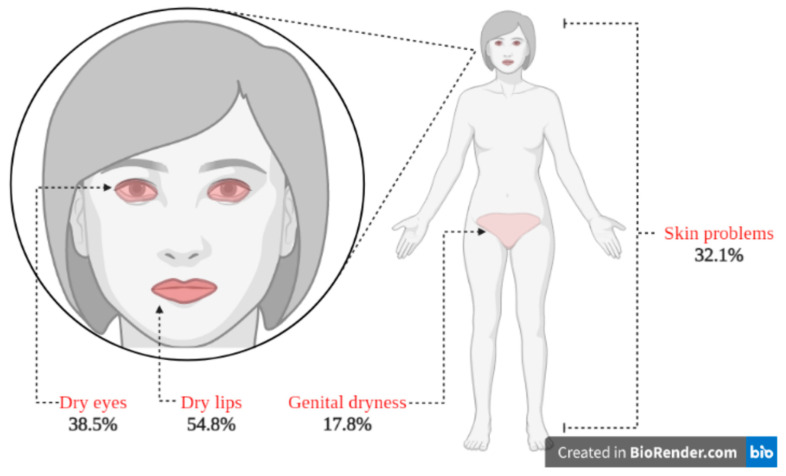
Extraoral dryness with location and prevalence (in percentage).

The most prevalent extraoral dryness in the BMS patients was located on the lips (54.8%), followed by the following order: in the eyes (38.5%), on the skin (32.1%), and in the genital area (17.8%). Figure 2 presents the location of extraoral dryness identified from the included studies.

## 4. Discussion

The objective of this systematic review was to identify and evaluate extraoral dryness in BMS. We hypothesized that dryness is not limited to intraoral dryness and can be found in other regions of the body. Our results confirmed this hypothesis, and the outcome showed the presence and distribution of extraoral dryness in BMS patients in the eyes, skin, lips, and genital area. In the following, the systematic review findings are discussed in detail.

### 4.1. Demographic Characteristics

A noticeable finding came from the comparison between the oldest (1987) [25] and the newest (2022) [43] studies in this review that showed slow and small progress in clinical knowledge about BMS. This was mainly reflected in the clinical diagnosis and extraoral characteristics of BMS. The diagnostic criteria for BMS patients were different between the included studies. This variation comes from different definitions of BMS used in the studies. Most studies used a predefined BMS definition for their clinical diagnosis. The most used (Study 3 [43], Study 6 [26], Study 7 [45], and Study 9 [47]) definition of BMS is the one given by the International Classification of Headache Disorders, 3rd Edition (ICHD-3 clinical definition: 13.11) [7], which defines BMS as an intraoral burning or dysesthesia sensation recurring daily for more than 2 h for at least 3 months without clinically evident causative lesions. Another definition for BMS (used by Study 4 [44]) is the one given by the International Classification of Diseases (ICD-10-CM diagnosis code K14.6) [48], which defines BMS as an intraoral burning or dysesthesia sensation daily for more than 2 h for at least 3 months in an otherwise clinically normal oral mucosa. Both of these particularly address the same criteria of either intraoral burning sensation or dysesthesia sensation in addition to an identical timeframe. The rest of the studies had vastly different definitions of BMS. Of those, Study 1 [25], Study 2 [42], and Study 8 [46] had importance for oral burning sensation, with Study 1 [25] and Study 8 [46] including a timeframe for occurrence for more than 3 months and more than 6 months, respectively, while Study 2 [42] did not have any time frame. This lack of a standardized timeframe could potentially influence the inclusion of different populations with different characteristics and therefore limit the ability to compare the results [50]. Study 5 [4] had a unique diagnosis for BMS, which was defined as chronic oral pain persisting daily for more than 6 months in individuals older than 18 years of age without any causative lesions in an otherwise clinically normal oral mucosa. This clinical diagnosis for their BMS group was different in that it included the age of the patients, as well as having both causative lesions and normal oral mucosa.

The lack of a standardized clinical diagnosis could have implications for the reliability and validity of the results of the individual-included studies, as the differences between the diagnoses could potentially introduce bias as well as inconsistent study results. Improvements in standardizing clinical diagnosis would improve the accuracy of the conclusions and increase confidence in the results [51]. In addition, the clinical characteristics are mainly limited to the intraoral characteristics for BMS with less or no inclusion of extraoral characteristics, including extraoral dryness.

The age group of BMS patients observed in the included studies (~57 to ~68 years) aligned with the typical age noted in BMS diagnostic criteria. We also identified that the majority of BMS patients were women. The age and sex characteristics of BMS hint at two main points: (1) age and body changes that could include the susceptibility to dry skin by the nature of decreased water content and overall drier skin [52] and (2) hormonal changes during the menopausal years of the affected women [53]. Lower estrogen levels could lead to not only skin dryness and thinning but also other skin concerns, such as decreased skin elasticity, firmness, wrinkles, and age spots [54]. Although these changes are more common in women experiencing menopausal changes, both men and women [55] experience skin changes due to several other factors, such as the aging process itself, individual lifestyle habits, and geographical location [56]. The latter poses a complex effect on body dryness, with some studies suggesting that living in drier, colder, and more arid environments affects skin barrier function and transepidermal water loss [57,58]. Our results showed that the included studies were conducted in the northern hemisphere except for Study 8 [46], which was conducted in Brazil, in the southern hemisphere. The northern hemisphere tends to have more seasonal changes that could include drier conditions in the winter months and, as such, would have lower humidity levels and colder temperatures that could lead to drier skin due to worsening skin surface hydration in these conditions [59]. In contrast, the southern hemisphere tends to have consistent humid weather throughout the year, which can help keep the skin hydrated. A comprehensive assessment of geographical location regarding the prevalence of extraoral dryness in BMS patients could not be extensively conducted due to the lack of prioritization in reporting seasonal variations, environmental factors, humidity levels, and temperature extremes specific to the locations of the included studies. As a result, it is challenging to draw definitive conclusions about the influence of geographical location on the occurrence of extraoral dryness in BMS patients based on the available data. In future BMS studies [19], therefore, considering the factors of seasonal variations, environmental factors, humidity levels, and temperature extremes, individuals living in different regions are encouraged.

### 4.2. Extraoral Dryness

Collectively, the included studies reported the presence of extraoral dryness. The bodily locations of dryness included the eyes, lips, genital regions, and skin. However, the extent of extraoral dryness was less investigated or reported in the included studies. This might be due to the lack of a standard methodology for data collection from BMS outside the mouth or focusing on intraoral characteristics. Some of the included studies had reported these characteristics with various terminologies such as “associated symptoms for BMS patients” or “comparisons of general health between BMS patients and control groups”. Therefore, these categories were not accessible to explore further, and the outcomes were merely utilized to present the existence of associated symptoms rather than detailed characteristics. Except for one study (Study 8 [46]), none of the other included studies had discussed their findings of extraoral dryness. This can be interpreted as less emphasis on the extraoral dryness in BMS patients, an unknown phenomenon for some investigators, or a less explored area of research where the importance is not yet fully understood. Nevertheless, we could extract some of the extraoral characteristics that were mentioned or explained in the included studies, even though those were far from an optimal or comprehensive presentation.

Out of nine studies, dry eyes were reported in five studies, dry lips were reported in two studies, genital dryness was reported in two studies, and skin problems were reported in three studies. These findings point to the fact that a standard reporting scheme in BMS is lacking in including both the intraoral and extraoral characteristics of dryness. Considering the evidence found through this systematic review, it is proposed that dryness must be added to the armamentarium of BMS clinical data collection. Even though self-reporting is an acceptable form of data collection, similar to what was collected in Study 4 [44], the preparation of a checklist or questionnaire can be very helpful for harmonizing data collection, similar to what was carried out in Study 6 [26], and Study 8 [46] by the aid of questionnaires. It is also important to consider and report the type of BMS, for example, primary and secondary, as it might influence the extraoral dryness characteristics [12]. Study 8 [46] was the only study that reported the type of BMS. Due to a lack of sufficient information, the association between the type of BMS and the pattern of extraoral dryness remains inconclusive.

Due to the lack of reported values for extraoral dryness in the included studies, the analysis was mainly limited to the description and presentation of the extracted characteristics. The outcome variables of the included studies were not sufficient to permit conducting a meta-analysis; therefore, we could only utilize analysis of the odds ratios for the occurrence of extraoral dryness in BMS patients and the control groups. Odds ratios are commonly used in epidemiology to address the strength of association between two variables [60,61]. The comparison between two populations (for example, patients and controls) and the ratio of the probability of an event of interest (for example, dryness) occurring in each population [62] are common in epidemiological studies. Extraoral dryness outcomes that were reported for the BMS patients’ group and control group, therefore, were statistically relevant, and we calculated odds ratios and their 95% confidence interval. The findings showed that the odds of extraoral dryness were largely higher in BMS patients compared with the control group. This means that dryness might be a more widespread characteristic in BMS patients that occurs intraorally and extraorally compared with the healthy controls. However, the wide confidence interval for the odds ratios marked the low precision of the estimates and the high variability in the data, which would entail a large degree of uncertainty. This was somewhat expected since the extraoral dryness values are not among clinical diagnoses as a potential BMS characteristic. Nevertheless, the finding of higher odds in BMS patients compared with the control group remains a valid point of interest. There are possibly other factors, such as genetics or environmental exposure, which could contribute to the differences observed between the patient and control groups.

Extraoral dryness not only complicates the features of the disease and the management of BMS but can dramatically impact the quality of life in the affected patients. This was, in fact, the focus of one of the studies in this systematic review (Study 7 [45]) which explored quality of life, related to the frequency of xerostomia-associated symptoms. The ratings range from never occurring (score 1) to very frequently occurring (score 5). They found a higher frequency of dry eyes, dry lips, and a dry nasal cavity for BMS patients compared with the control groups, although skin problems showed a relatively similar frequency rating between the BMS patients and control groups.

Study 8 [46] and Study 9 [47] only presented the outcomes for BMS patients and did not check the extraoral dryness of the control group; hence, the question remains open as to whether the prevalence of extraoral dryness would be statistically different between BMS patients and healthy controls in these two studies and whether they could influence the overall outcome of this systematic review. Generally, it is expected that there will be further exploration in larger sample size studies of extraoral dryness in BMS. It is recommended to include proper control groups in future studies as dryness also occurs in normal healthy populations.

Related to regions where the extraoral dryness in BMS occurred, we identified dry eyes with the lowest odds, skin problems with odds that were in the middle of the spectrum, and genital dryness with the highest odds. Dry lips, however, did not have a comparator and, therefore, were not included in the analysis. Collectively, our findings show that BMS patients have higher odds of extraoral dryness and that the dryness occurs in different regions of the body, with the highest odds for the genital area. Although the occurrence odds of genital dryness are the highest, it is interesting to note that it has the lowest percent prevalence among BMS patients. This suggests that while genital dryness may occur more frequently compared to the other regions, it is not as commonly reported by a significant portion of BMS patients or this feature might not be commonly assessed by clinicians or professionals during clinical evaluation. Instead, symptoms such as dryness of the lips, eyes, or skin appear to be more prevalent. These findings emphasize the need for a comprehensive approach to address extraoral symptoms in BMS patients. Addressing dryness in the identified regions is crucial to offer help for subsiding and improving the overall quality of life for BMS patients. This finding is important in the clinical management of BMS patients, who are mainly treated for intraoral disturbances. We, therefore, draw attention to the extraoral symptoms, namely, extraoral dryness, that can be disturbing to patients.

Potential mechanisms underlying extraoral dryness in BMS have not been investigated. Various hypotheses have emerged [6,63] related to neurological, endocrinological, and hormonal contributions in BMS and its comorbid conditions (e.g., depression, anxiety, dementia, and Parkinson’s disease) [64] but not for extraoral dryness. Numerous factors have also been mentioned to act as confounding factors, such as age, sex, lifestyle, environmental factors, other disorders, medication use, and genetic predisposition. Further investigation into extraoral manifestations of BMS, therefore, helps in understanding the underlying mechanisms, provides updates on relevant diagnostic criteria, and helps in developing evidence-based treatment approaches. Notably, most of the treatments target intraoral manifestations [65]. A 2017 systematic review of BMS treatments presents the application of alpha-lipoic acid, clonazepam, capsaicin, and gabapentin [65]. A 2021 systematic review of nonpharmacologic therapies for BMS [66] presents 14 different interventions, including acupuncture, the application of tocopherol, catuama, and ultramicronized palmitoylethanolamide, cognitive therapy and group psychotherapy, and prefrontal cortex repetitive transcranial magnetic stimulation (rTMS) [66]. Despite the variety of therapies used to treat BMS, the outcomes assessed usually refer to intraoral symptoms.

### 4.3. Other Findings

The other associated symptoms that were reported by the included studies were not uniform. The most common associated symptom was xerostomia. Xerostomia [67,68], or the sensation of dry mouth, is the most commonly reported symptom of BMS. The underlying mechanisms of dry mouth have been linked to several factors. For example, a significant reduction in salivary function and salivary composition in older patients has been reported [69,70]. Hormonal changes in menopausal women [71] and changes in the oral cavity [72] are also reported to be associated with the sensation of oral dryness. Some studies also suggest that dry mouth may lead to BMS. For example, xerostomia, secondary to the consumption of amlodipine, has been reported as an underlying cause of BMS [73].

Other examples of the most commonly reported symptoms are gastrointestinal problems, dysgeusia, and insomnia. Only one study reported the type of gastrointestinal problem as gastroesophageal reflux disease in Study 4 [44]. Lamy et al. [42] showed an association between BMS and gastrointestinal problems. Gastrointestinal problems can be related to lower salivary flow [74] or a consequence of stress and anxiety [75] that often accompany BMS [12]. Dysgeusia (metallic or distorted taste) was also identified in this systematic review and has also been reported in the literature [76]. Dysgeusia is generally a common symptom reported with various oral disorders, such as oral lichen planus, candidiasis, and taste disorders [77]. Hormonal changes in the menopausal period have been reported to be associated with dysgeusia [78]. Insomnia might be related to difficulty sleeping as a consequence of the general discomfort experienced by patients with BMS [79]. The stress and anxiety that accompany BMS might also exacerbate sleep problems [80]. Insomnia is also a general problem among the elderly [81,82]. It is reported that older women are more likely to suffer from insomnia [83]. Hormonal changes in the menopausal period are also reported to be associated with sleep disorders, including insomnia [84].

Candidiasis was reported in Study 9 [47], which is a condition commonly reported in the literature as an associated symptom in oral lesions and some oral disorders. According to a majority of reports, BMS often appears without any obvious lesions or laboratory test abnormalities [6]. No direct association has been found between the presence of *Candida* species and BMS [85].

Taken together, the presence of various symptoms in BMS implies that the mechanisms underlying BMS might influence a broader spectrum than the intraoral cavity. Indeed, brain imaging studies have demonstrated structural and functional changes in BMS compared with healthy individuals [86]. We speculate that at least three potential mechanisms can be investigated in the future to explain the extraoral dryness: (1) Dryness can directly cause pain and pain can exacerbate dryness, most likely intraorally. Under a persistent and long-term intraoral dryness–pain interaction, central sensitization of pain and a more generalized immune reaction may occur that can manifest in a larger spread of dryness and perhaps higher responses in somatosensation, for example, allodynia to touch extraorally. (2) Dryness is a comorbid condition in BMS and it is not directly or indirectly linked to pain. This can be due to a widespread reaction in the immune system to BMS. (3) Since cases of BMS without dryness also exist, there must be other phenomena or mechanisms involved, which remain unknown. This latter hypothesis needs further investigation to determine whether it is caused by genetic, epigenetic, or a combination of both potential factors.

Regardless of the mechanism, the clinical value of extraoral symptoms is to urge healthcare professionals to develop more comprehensive diagnostic criteria and treatment approaches tailored to the specific needs of BMS patients. This will consequently help patients’ care, lowering the burden and enhancing quality of life.

## 5. Strength and Limitations

We conducted a systematic review to provide a comprehensive and unbiased synthesis of existing evidence on extraoral dryness in BMS. Systematic reviews have been reported to be valuable resources for evidence synthesis and, accordingly, for evidence-based decision-making and policy formulation [87]. We, therefore, consider the choice of this review type as a strong point. Although the sample size was small, the quality assessment of the included articles demonstrated a fair level of quality and allowed the narrative synthesis to identify an overall outcome about extraoral dryness in BMS. Therefore, the findings can be used to inform future research directions, clinical practice, and the development of tailored interventions for individuals affected by BMS.

Several limitations, however, are present. The inclusion of EBSCOhost resulted in several duplicate studies in the literature search phase. EBSCOhost has multiple databases, including MEDLINE, which is a large subset of PubMed. Language bias might have limited the included articles since only English-language studies were considered. Considering the high level of evidence that is often provided by randomized clinical trials, we only included case–control and case series studies, which might be susceptible to bias, which can impact result accuracy. These study designs are generally considered lower-quality evidence compared to randomized control trials and cohort studies, according to the evidence pyramid [88,89]. The limitations are mainly due to the design of these studies, which prevents causality identification and examination of the influence of confounding factors [90].

Another limitation is related to extraoral dryness when it comes to the skin. Differences between the studies reporting skin problems with limited information about location and characteristics led to the consideration of dry skin as an overall patient complaint rather than a specific condition.

Using narrative synthesis in a systematic review has drawbacks, including the increased risk of bias, limited data comparability, and a potential lack of transparency [91,92]. Here, a narrative synthesis was used, using odds ratios to assess the association of extraoral dryness in BMS patients. However, this methodology does not fully measure absolute risk or event probability [92]. A point worth mentioning is that Study 4 [44] had extraoral dryness values for both dry eyes and genital dryness from the same population group, but this study did not address which individuals had what values [44]. This means that those individuals who had both dry eyes and genital dryness could increase the rate of overall dryness in the summed population group, which brings into question the distribution and impact of both dry eyes and genital dryness.

## 6. Conclusions and Future Perspectives

Extraoral dryness in BMS patients has been largely neglected. The findings from this systematic review indicated that extraoral dryness occurs in BMS patients with higher odds than non-BMS controls. The pattern of occurrence of extraoral dryness in different body regions indicated a potential systemic origin of dryness that expands beyond the intraoral cavity in BMS. The most prevalent sites were—from most to least—dry lips, dry eyes, dry skin, and dry genital areas. There is a clear need to include standardized methods to evaluate and document extraoral clinical manifestations of BMS, such as extraoral dryness. These manifestations can also be added to diagnosis criteria or accompanying symptoms to help clinicians better understand and manage BMS. This would, in turn, facilitate and enhance the quality of patient care. Currently, no definitive guideline exists for handling extraoral dryness in BMS. More research is also needed to understand the pathomechanisms underlying extraoral dryness in BMS, including exacerbating risk factors. This can be performed with the aid of preclinical and clinical studies. Campaigns for patient awareness and education about BMS and its accompanying symptoms seem useful for empowering patients and increasing their health literacy to report their symptoms to clinicians and seek advice for preventing or treating extraoral problems in BMS.

## Figures and Tables

**Table 1:** PICO model for clinical questions.

Population	Intervention	Comparison	Outcome
Burning mouth syndrome patients (aged women)	Not applicable	Extraoral dryness and what type/to which degree Comorbidities Diagnostic criteria	Exploring the etiology of burning mouth syndrome by investigating to which degree extraoral dryness is found

**Table 2:** Inclusion and exclusion criteria.

Criteria	Inclusion	Exclusion
Population	● BMS patients ● Aged women	● Men, children, and teens ● Animal studies and in vitro studies
Study design	● Observational studies ● Experimental studies	● Abstracts ● Non-full text journals ● Opinion papers ● Letters
Data/outcome	● Extraoral dryness in BMS ● Comorbidities in BMS ● Empirical quantitative data	● Qualitative data

**Table 3:** The search terms and databases for the literature search.

Databases Searched	Search Terms
Google Scholar	1. “Burning mouth syndrome”|“Burning mouth disorder” AND prevalence “Dry skin”|“Xerosis”|“Skin disease” body|“body region” 2. “Burning mouth syndrome” OR “Burning mouth disorder” AND prevalence “Dry skin” OR “Xerosis” OR “Skin disease” AND “body” OR “body region” 3. “Burning mouth syndrome” OR “Burning mouth disorder” AND prevalence “Dry skin” OR “Xerosis” OR “Dermatitis” OR “Skin disease” AND “body” OR “body region” 4. “Burning mouth syndrome patients” OR “BMS patients” AND prevalence “Dry skin” OR “Xerosis” OR “Dermatitis” OR “Skin disease” AND “body” OR “body region” 5. “Burning mouth syndrome patients” AND “dryness” AND “association”
PubMed	1. (“Burning mouth syndrome patients” OR “BMS patients”) AND (Dry skin OR Xerosis OR Dermatitis OR Skin disease) 2. (“Burning mouth syndrome patient*” OR “BMS patient*”) AND (Association) 3. (“Burning mouth syndrome patient*” OR “BMS patient*”) AND (“comorbidit*”)
Web of Science	1. (“Burning mouth syndrome patient*” OR “BMS patient*”) (Topic) and (Dry skin OR Xerosis OR Dermatitis OR Skin disease*) (Topic) 2. (“Burning mouth syndrome patient*” OR “BMS patient*”) (Topic) and (“Comorbidit*”) (Topic)
COCHRANE Library	1. (“Burning mouth syndrome patient*”) OR (“BMS patient*”) AND (Comorbidit*)
EBSCOhost	1. (((“burning mouth syndrome”) OR (“BMS”) AND patient*)) AND (comorbidit*) 2. (((“burning mouth syndrome”) OR (“BMS”) AND patient*)) AND (Dry skin OR Xerosis OR Dermatitis OR Skin disease*)

**Table 4:** Diagnostic criteria used by the included studies.

Study ID	Diagnostic Criteria for BMS	Diagnostic Criteria for the Control Groups
Study 1 [25]	“Complaint of persistent oral burning for >3 months in an otherwise clinically normal oral mucosa”	Sex- and age-matched participants
Study 2 [42]	“Burning sensation in an otherwise clinically normal oral mucosa”	Sex-, age-, marital-, and employment status-matched participants with no prior diagnosis of BMS
Study 3 [43]	ICHD-3 clinical definition: 13.11	Participants who did not meet the ICHD-3 clinical definition: 13.11 diagnostic criteria
Study 4 [44]	ICD-10-CM diagnosis code K14.6	Participants with no prior diagnosis of BMS or other oral complications
Study 5 [4]	“Participants of >18 years old with chronic oral pain daily for >6 months, without clinically evident causative lesions in an otherwise clinically normal oral mucosa”	>18-year-old participants without evident oral lesions of unexplained oral symptoms
Study 6 [26]	ICHD-3 clinical definition: 13.11	Age-matched women who did not meet the ICHD-3 clinical definition: 13.11 diagnostic criteria
Study 7 [45]	ICHD-3 clinical definition: 13.11	Sex- and age-matched participants who did not meet the ICHD-3 clinical definition: 13.11 diagnostic criteria
Study 8 [46]	“Burning sensation for >6 months, without clinically evident causative change”	Sex- and age-matched participants without oral burning or lesions
Study 9 [46]	ICHD-3 clinical definition: 13.11	Not applicable

**Table 5:** Assessment of risk of bias within the case–control studies.

Study ID	Q1	Q2	Q3	Q4	Q5	Q6	Q7	Q8	Q9	Q10	Q11	Q12	Overall Risk of Bias
Study 1 [25]	Y	Y	N	Y	Y	Y	N	Y	N	N	N	N	Fair (6)
Study 2 [42]	Y	Y	N	Y	Y	N	Y	Y	Y	Y	N	Y	Fair (9)
Study 3 [43]	Y	Y	N	Y	Y	Y	Y	Y	Y	Y	N	N	Fair (9)
Study 4 [44]	Y	Y	N	Y	Y	Y	Y	Y	Y	Y	N	N	Fair (9)
Study 5 [4]	Y	Y	N	Y	Y	Y	Y	Y	Y	Y	N	N	Fair (9)
Study 6 [26]	Y	Y	N	Y	Y	Y	Y	Y	N	N	Y	N	Fair (8)
Study 7 [45]	Y	Y	N	Y	Y	Y	N	N	Y	Y	N	N	Fair (7)
Study 8 [46]	Y	Y	N	Y	Y	Y	N	Y	Y	Y	N	N	Fair (8)

**Table 6:** Assessment of risk of bias within the case series studies.

Study ID	Q1	Q2	Q3	Q4	Q5	Q6	Q7	Q8	Q9	Overall Risk of Bias
Study 9 [47]	Y	Y	Y	N	N	Y	Y	N	Y	Fair (6)

**Table 7:** Description of the study characteristics of the included studies.

Study ID	Study Title	Year Published	Place of Origin	Study Design	Aims
Study 1 [25]	Clinical features of burning mouth syndrome	1987	Canada	Case–control	Comparison of clinical features between BMS patients and age- and sex-matched control subjects
Study 2 [42]	Vulnerability and presenting symptoms in burning mouth syndrome	2005	UK	Case–control	Comparison of clinical features and health quality between BMS patients and age- and sex-matched control subjects, as well as to examine the role of vulnerability factors and differentiate them from presenting symptomology in patients with BMS
Study 3 [43]	Central sensitization in burning mouth syndrome: a practical approach using questionnaires	2022	Spain	Case–control	Comparison of central sensitization presence between BMS patients and age- and sex-matched control subjects, as well as measuring the extent of pain, the presence of associated symptoms, and other chronic pain conditions
Study 4 [44]	Saliva flow rates and clinical characteristics of patients with burning mouth syndrome: a case–control study	2021	USA	Case–control	Comparison of unstimulated and stimulated saliva flow rates, mucosal hydration, and xerostomia between BMS patients and age- and sex-matched control subjects
Study 5 [4]	Unexplained somatic comorbidities in patients with burning mouth syndrome: a controlled clinical study	2011	Italy	Case–control	Comparison of unexplained extraoral symptoms prevalence in BMS patients, oral lichen planus patients, and age- and sex-matched control subjects
Study 6 [26]	Clinical characterization of women with burning mouth syndrome in a case–control study	2018	Sweden	Case–control	Comparison of underlying factors, clinical characteristics, and patients’ self-reported oral and general health factors associated between BMS patients and age- and sex-matched control subjects
Study 7 [45]	General health status of a sample of patients with burning mouth syndrome: a case–control study	2020	Spain	Case–control	Comparison of general health between BMS patients and age- and sex-matched control subjects
Study 8 [46]	Burning mouth syndrome: clinical profile of Brazilian patients and oral carriage of Candida species	2007	Brazil	Case-control	Comparison of clinical profile between BMS patients and age- and sex-matched control subjects
Study 9 [47]	Burning mouth syndrome: a diagnostic challenge	2020	USA	Case series	To characterize the diagnostic process for BMS patients and identify pitfalls encountered in the workup and management of BMS

**Table 8:** Characteristics of the participants in the included studies.

Study ID	Participants	Sex	Age	Diagnostic Criteria (BMS)	Diagnostic Criteria (Control)
Study 1 [25]	BMS n = 54 Control n = 27	BMS and Control 84/102 women (82%)	BMS and Control Weighted mean of 57.88	Complaint of persistent oral burning for >3 months in an otherwise clinically normal oral mucosa	Sex- and age-matched participants
Study 2 [42]	BMS n = 84 Control n = 73	BMS and Control 138/160 women (88%)	BMS and Control Mean of 65	Burning sensation in an otherwise clinically normal oral mucosa	Sex-, age-, marital-, and employment status-matched participants without prior experience of burning mouth syndrome or cognitive impairment
Study 3 [43]	BMS n = 40 Control n = 42	BMS 37/40 women (92.5%) Control 39/42 women (92.9%)	BMS Weighted mean of 61.96	ICHD-3 clinical definition: 13.11: Intraoral burning or dysesthesia sensation recurring daily for >2 h for >3 months without clinically evident causative lesions	Participants who did not meet the ICHD-3 clinical definition: 13.11 diagnostic criteria
Study 4 [44]	BMS n = 22 Control n = 28	BMS All women (100%) Control All women (100%)	BMS Weighted mean of 58.64	ICD-10-CM diagnosis code K14.6: Intraoral burning or dysesthesia sensation daily for >2 h for >3 months in an otherwise clinically normal oral mucosa	Participants with no prior diagnosis of BMS and who answered specific questions regarding oral disturbances in the past 3 months
Study 5 [4]	BMS n = 124 Control n = 102	“Mostly” women *	BMS Weighted mean of 57.23	Participants of >18 years old with chronic oral pain daily for >6 months without clinically evident causative lesions in an otherwise clinically normal oral mucosa	Participants of >18 years old without evident oral lesions or unexplained oral symptoms, no history of psychiatric disorder, and first-time consultation in such a study
Study 6 [26]	BMS n = 56 Control n = 56	BMS All women (100%) Control All women (100%)	BMS Weighted mean of 67.75	ICHD-3 clinical definition: 13.11: Intraoral burning or dysesthesia sensation recurring daily for >2 h for >3 months without clinically evident causative lesions	Age-matched women who did not meet the ICHD-3 clinical definition: 13.11 diagnostic criteria, and other oral mucosal changes, infections, and illnesses
Study 7 [45]	BMS n = 20 Control n = 40	BMS 16/20 women (80%) Control 32/40 women (80%)	BMS Weighted mean of 63.72	ICHD-3 clinical definition: 13.11: Intraoral burning or dysesthesia sensation recurring daily for >2 h for >3 months without clinically evident causative lesions	Sex- and age-matched participants, who did not meet the ICHD-3 clinical definition: 13.11 diagnostic criteria
Study 8 [46]	BMS n = 31	BMS 28/31 women (90.3%)	BMS Mean of 61.3	Burning sensation for >6 months, without clinically evident causative change	Sex- and age-matched participants without burning complaints and without oral lesions
Study 9 [47]	BMS n =102	BMS 88/102 women (86.3%)	BMS Median of 60	ICHD-3 clinical definition: 13.11: Intraoral burning or dysesthesia sensation recurring daily for >2 h for >3 months without clinically evident causative lesions	No comparator

* = the number of females and males was not reported.

**Table 9:** Details of the main outcomes of the included studies.

Study ID	Extraoral Dryness Outcomes for BMS	Extraoral Dryness Outcomes for Control	BMS Types	Reported Associated Symptoms	Conclusions
Study 1 [25]	Dry eyes 18/54 (33.3%)	Dry eyes 4/27 (14.8%)	Not reported	Dysgeusia Insomnia Xerostomia	The study found little evidence to support earlier theories that dentures, oral habits, and nutritional deficiencies are significant causes of burning mouth syndrome (BMS), although some differences were found between the BMS and control subjects. However, the study suggested that changes in salivary composition might be linked to the subjective xerostomia prevalent in BMS subjects. Compared to the control subjects, the BMS subjects reported a higher prevalence of symptoms such as dry mouth, thirst, taste and sleep disturbances, headaches, nonspecific health problems, pain complaints, and severe menopausal symptoms but had no differences in other oral or dental features or the prevalence of candidiasis infection.
Study 2 [42]	Skin problems 23/84 (27.3%)	Skin problems 7/73 (9.6%)	Not reported	Carcinophobia Chronic fatigue Circadian rhythm disorders Dizziness Gastrointestinal problems Insomnia Rheumatoid arthritis Skin alterations Somatic complaints **	The study reports patients with BMS had poorer overall health, more illnesses and gastrointestinal problems, chronic fatigue, disturbed sleep patterns, and more anxiety and depression compared to control subjects. The study proposes that perceptions of ill health may be related to presenting symptoms and life experiences and that vulnerability factors may be associated with experiencing emotional distress as a bodily illness. The study recommends considering vulnerability factors as potential markers for those who experience emotional distress as bodily illness in BMS patients.
Study 3 [43]	Dry eyes 19/40 (47.5%)	Dry eyes 11/42 (26.2%)	Not reported	Cognitive impairment Common mental illnesses Dysgeusia Dizziness Insomnia Myasthenia Xerostomia	The study results suggest the presence of CS in BMS patients, and the study proposes that evaluating the degree of CS is useful for complementing clinical diagnosis and aiding therapeutic decision-making. The study found that patients with BMS had significantly higher scores for nonrestorative sleep, sleep disorders, fatigue, and anxiety than the controls and that these factors should be considered in the management of patients with BMS. Symptoms related to BMS and those associated with CS both obtained high scores in patients with BMS, suggesting that the list of comorbidities closely associated with BMS is a useful tool for identifying this disorder.
Study 4 [44]	Dry eyes 12/22 (54.5%) Genital dryness 11/22 (50%)	Dry eyes 11/28 (39.3%) Genital dryness 5/28 (17.8%)	Not reported	Gastrointestinal problems Genital dryness	The study of burning mouth syndrome (BMS) in older women found that BMS cases had lower USWS flow rates and higher prevalence of xerostomia, vaginal dryness, and GI disease compared to controls. The study found a significant difference in the mean USWS flow rate in cases compared to controls, suggesting that the sensation of oral dryness is correlated with a reduction in salivation. Medication usage is possibly a contributing factor in BMS patients using more than one medication. Gastrointestinal disease was more commonly reported among BMS cases than the controls. Vaginal dryness was significantly more common among BMS cases. The study had several strengths and limitations, including a well-characterized sample of cases and controls.
Study 5 [4]	Genital dryness 15/124 (12.1%)	Genital dryness 0/130 (0%)	Not reported	Gastrointestinal problems Globus sensation Myofascial pain Ocular burning Otorhinolaryngological problems Palpitations Tension-type headaches Tinnitus	The study aimed to assess the prevalence of unexplained extraoral symptoms in patients with burning mouth syndrome (BMS) compared to patients with oral lichen planus (OLP) and healthy individuals. The study collected data from 124 BMS patients, 112 OLP patients, and 102 healthy patients. The results showed that 96.1% of BMS patients reported unexplained extraoral symptoms compared to 9.3% in OLP patients and 15.7% in healthy individuals. BMS patients reported painful symptomatology in different bodily regions more frequently than OLP patients and healthy individuals. The study suggests that BMS may be classified as a complex somatoform disorder rather than a neuropathic pain entity and that various medical disciplines should be involved in the diagnostic process.
Study 6 [26]	Skin problems 22/56 (39.3%)	Skin problems 8/56 (14.3%)	Not reported	Chronic pain Xerostomia	The study compared patients with BMS and age-matched controls. Skin diseases were strongly associated with BMS, as were self-reported symptoms of xerostomia. The groups did not differ with respect to background factors. The BMS group reported poorer general and oral health and poorer life satisfaction than the controls. Hormonal status may play a role in the pathogenesis, but studies on hormone replacement therapy show contradictory results. Patients with BMS reported more allergies and medications than controls. Bruxofacets were more common in patients with BMS but did not contribute to the regression model.
Study 7 [45]	Xerostomia Inventory * Skin problems 1.85 ± 1.31 Dry eyes 3.70 ± 0.92 Dry lips 4.05 ± 0.83 Dry nose cavity 2.70 ± 1.30	Xerostomia Inventory * Skin problems 1.85 ± 1.05 Dry eyes 1.65 ± 0.89 Dry lips 2.23 ± 1.05 Dry nose cavity 1.98 ± 0.85	Not reported	Asthma Chronic pain Fibromyalgia Osteoarthritis Sleepiness Xerostomia	The study reports that burning mouth syndrome (BMS) patients suffer from more comorbidities and consume more medications than controls. Mental, behavioral, or neurodevelopmental disorders were found more frequently in BMS patients, who also used four times more drugs, especially for the nervous and cardiovascular systems, as well as the alimentary tract and metabolism. BMS patients had lower iron and higher folic acid levels than the controls, and their general health status, oral health impact, sleepiness, psychological status, and xerostomia levels were significantly worse. BMS is usually associated with mental and behavioral disorders like depressive and anxiety disorders, making the positive correlation in the use of psycholeptics and psychoanaleptics logical.
Study 8 [46]	Dry lips 17/31 (54.8%)	No relevant comparator	Type 1 (48.4%) Type 2 (35.5%) Type 3 (16.1%)	Carcinophobia Xerostomia	The study reports that the majority of BMS patients were postmenopausal women with chronic medication use, particularly antihypertensives and antidepressants, and secondary complaints such as dry mouth and altered taste. Anemia and prosthetic adjustments did not significantly affect BMS symptoms. The study found positive cultures for *Candida* species in 45.16% of patients, but this did not confirm the presence of *C. albicans* as an associated factor in BMS etiology. The study highlights the importance of interdisciplinary investigation and patient education to address psychological factors and lifestyle changes associated with BMS.
Study 9 [47]	Dry eyes 35/102 (34.3%)	No comparator	Not reported	Candidiasis *** Chronic pain, Common mental illnesses Dysgeusia Hyposalivation Xerostomia	The study reports that burning mouth syndrome (BMS) poses a diagnostic challenge and causes significant delays in diagnosis, often with patients seeing multiple providers before presenting to an oral medicine specialist. Misdiagnosis is common, with candidiasis being the most frequent provisional diagnosis. Patients often present with extraoral comorbidities, including anxiety and depression. BMS is underrecognized and underappreciated by both medical and dental professionals. A clinician with more familiarity with BMS can quickly diagnose and begin the proper management regimen without delay.

* = Skin problems described as “rosacea, eczema, dry skin and psoriasis”; ** = Somatic problems described as “dizziness, back pain, nausea”; *** = Candidiasis reported as “usually accompanied by lesions”.

**Table 10:** Extraoral dryness outcome in the included studies and calculated odds ratio.

Study ID	Extraoral Dryness Outcomes for BMS	Extraoral Dryness Outcomes for Controls	Odds Ratio (95% Confidence Interval)
Study 1 [25]	Dry eyes 18/54 (33.3%)	Dry eyes 4/27 (14.8%)	Dry eyes 2.88 (0.86, 9.58)
Study 2 [42]	Skin problems 23/84 (27.4%)	Skin problems 7/73 (9.6%)	Skin problems 3.56 (1.43, 8.88)
Study 3 [43]	Dry eyes 19/40 (47.5%)	Dry eyes 11/42 (26.2%)	Dry eyes 2.55 (1.01, 6.44)
Study 4 [44]	Dry eyes 12/22 (54.5%) Genital dryness 11/22 (50%)	Dry eyes 11/28 (39.3%) Genital dryness 5/28 (17.8%)	Dry eyes 1.85 (0.60, 5.75) Genital dryness 4.60 (1.28, 16.51)
Study 5 [4]	Genital dryness 15/124 (12.1%)	Genital dryness 0/130 (0%)	NA
Study 6 [26]	Skin problems 22/56 (39.3%)	Skin problems 8/56 (14.3%)	Skin problems 3.88 (1.55, 9.75)
Study 7 [45]	Xerostomia Inventory * Skin problems 1.85 ± 1.31 Dry eyes 3.70 ± 0.92 Dry lips 4.05 ± 0.83 Dry nose cavity 2.70 ± 1.30	Xerostomia Inventory * Skin problems 1.85 ± 1.05 Dry eyes 1.65 ± 0.89 Dry lips 2.23 ± 1.05 Dry nose cavity 1.98 ± 0.85	NA
Study 8 [46]	Dry lips 17/31 (54.8%)	No data **	NA
Study 9 [47]	Dry eyes 35/102 (34.3%)	No comparator	NA

* = The Xerostomia Inventory consists of 14 questions that the participants would answer and the results for the quality of life ratings of xerostomia, reflecting the frequency of occurrence. A greater numerical value equates to a higher level of xerostomia reported. ** = Data of the control group were not reported. NA = not applicable.

**Table 11:** Summary of extraoral dryness values in BMS patients and control groups.

Summed Reported Extraoral Dryness Values	In BMS Patients	In the Control Group	Odds Ratio (95% Confidence Interval)
Dry eyes	$\frac{84}{218}$ = 38.5%	$\frac{26}{97}$ = 26.8%	1.7 (1.01, 2.88)
Genital dryness	$\frac{26}{146}$ = 17.8%	$\frac{5}{158}$ = 3.2%	6.6 (2.47, 17.78)
Skin problems	$\frac{45}{140}$ = 32.1%	$\frac{15}{129}$ = 11.6%	3.6 (1.89, 6.86)
Dry lips	$\frac{17}{31}$ = 54.8%	No comparator	Not applicable
Overall dryness	$\frac{172}{535}$ = 32.1%	$\frac{46}{384}$ = 12.0%	2.7 (1.88, 3.84)

## Data Availability

All data are available upon request.
